# Probiotics in the Treatment of Colorectal Cancer

**DOI:** 10.3390/medicines5030101

**Published:** 2018-09-07

**Authors:** Robert Hendler, Yue Zhang

**Affiliations:** Department of Hematology and Oncology, Stony Brook University Hospital, Stony Brook, New York, NY 11794, USA; yue.zhang@stonybrookmedicine.edu

**Keywords:** probiotics, synbiotics, microbiome, microbiota, colorectal cancer

## Abstract

The human microbiome plays many roles in inflammation, drug metabolism, and even the development of cancer that we are only beginning to understand. Colorectal cancer has been a focus for study in this field as its pathogenesis and its response to treatment have both been linked to the functioning of microbiota. This literature review evaluates the animal and human studies that have explored this relationship. By manipulating the microbiome with interventions such as probiotic administration, we may be able to reduce colorectal cancer risk and improve the safety and effectiveness of cancer therapy even though additional clinical research is still necessary.

## 1. Introduction

Globally, colorectal cancer is the third most commonly diagnosed type of cancer in men and the second most common in women [1]. In the United States, there are an estimated 135,430 new cases of colorectal cancer annually with an estimated 50,260 deaths due to the disease each year [2]. Colorectal cancer mortality has been improving over time in the United States by about 2.5% to 3% per year since 1990 [3] and while survival rates remain poor in advanced disease, early-stage colon cancer, this can potentially be cured with an estimated 74% five-year survival rate for patients with stage I disease [4]. As survival improves, it becomes more and more important to mitigate the adverse effects of colon cancer treatment. Surgery, chemotherapy, immune therapy, and radiation therapy all carry the possibility of significant treatment-related morbidity and mortality. In particular, adverse events affecting the gastrointestinal tract—such as nausea, diarrhea, colitis, and gastrointestinal bleeding—can diminish a patient’s quality of life, preclude additional treatment, and sometimes even become life-threatening.

One promising avenue of study in the prevention and treatment of colorectal cancer is with the human microbiota. This refers to the 10 to 100 trillion symbiotic microbial cells harbored by each individual largely located in the gut where they are involved with metabolizing food remnants, intestinal and digestive secretions, and exfoliated colonocytes. The human microbiome, in turn, refers to the genes contained by these cells [5]. The microbiota serves a myriad of roles in the body with both positive and negative effects on human health and several large ongoing projects exist to explore this complex interplay [6,7]. Complicating this research is the fact that the microbiome is extremely diverse between individuals. The entire human genome consists of approximately 22,000 genes. This is compared to the human gut microbiome, which contains 3.3 million non-redundant genes that vary between individuals [5].

In the context of this diversity, there is debate over what makes up a “healthy” human microbiome. When the total DNA content of microbes inhabiting humans is analyzed, only one third of their constituent genes are found in a majority of healthy individuals [8]. Early research into the human microbiome had focused on identifying a core set of microbial taxa that were universally present in healthy people who lacked overt disease phenotypes on the assumption that certain specific microbes were essential to health. However, this proved to be an oversimplification as the variety of microbial species between individuals meant that characterizing an ideal set of specific microbes was not practical. An alternative hypothesis is that a healthy microbiome represents a “functional core”—a set of metabolic and other molecular functions performed by the microbiome that are not necessarily performed by the same organism in every individual.

As research into the microbiome progresses, we find that it affects health in ways we wouldn’t expect. For instance, disruptions in the relative proportions of gut microbial populations may affect weight gain and insulin resistance contributing to type II diabetes mellitus [9]. These discoveries extend to the complex relationship between gut microflora, cancer development, and the effectiveness of cancer treatments. The composition of the microbiome has been correlated with colon cancer development in mouse [10,11] and human studies [12]. There is evidence that the effectiveness of various chemotherapy [13,14,15] and immune therapy [16,17] regimens depend on the composition of the microbiome. 

Manipulating the microbiome using probiotics to improve the safety and GI side effect profile of cancer treatment has also been explored with a few studies evaluating the possible clinical benefit of probiotics with surgery [18], radiation therapy [19], and chemotherapy [20]. Probiotics are attractive as a potential complement to treatment because they are inexpensive and associated with few, if any, major adverse effects. Available evidence also suggests that probiotics may have a significant clinical impact. For instance, in one study of 168 patients evaluated after colorectal surgery for cancer, those receiving probiotics had a significantly decreased rate of all postoperative major complications when compared to the placebo arm (28.6% vs. 48.8%, *p* = 0.010) [21]. However, prospective studies evaluating the clinical effect of probiotics in cancer patients are currently few in number and more evidence is needed to determine the situations in which probiotics are beneficial.

In addition, the composition of the gut microbiome is heavily dependent on diet. In one mouse study, switching from a low-fat, plant polysaccharide-rich diet to a high-fat, high-sugar “Western” diet had major effects on the microbiome within one day with measurable changes in microbiome metabolic pathways and gene expression [22]. Of particular interest are prebiotics, which are non-digestible food ingredients that benefit the host by stimulating the growth or activity of commensal bacteria in the gut [23]. These have been shown to significantly change the microbiome composition. Administration of fructo-oligosaccharides, for instance, significantly increases the number of bifidobacteria found on fecal analysis and it decreases the amount of *Bacteroides*, *Fusobacteria*, and *Clostridium* [24]. Furthermore, prebiotics such as long and short chain oligosaccharides undergo fermentation in the colon by anaerobic bacteria, which metabolize them to short chain fatty acids (SCFAs) such as acetate, propionate, and butyrate. An increased concentration of SCFAs in the colon seems to have several beneficial effects including improved barrier function, increased intestinal mucus synthesis, stimulation of immunosuppressive cytokines such as interleukin 10 (IL-10), and reduced levels of pro-inflammatory mediators. Furthermore, increased SCFAs seem to result in the preferential growth of protective bacteria over pathogenic strains [25]. Synbiotics—combinations of pre-biotics and probiotics—have been shown to have similar beneficial regulatory effects. 

## 2. Correlation between the Microbiome and Overall Gastrointestinal Health

**Figure 1:**
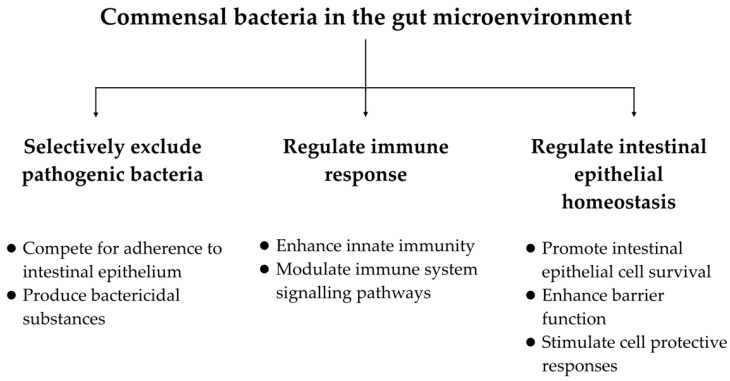
Proposed functions of gut commensal bacteria that may be beneficial in the prevention and treatment of colorectal cancer.

We know from a number of studies that the microbiome plays an integral role in gastrointestinal health and function and that manipulation of the microbiome—using probiotics, for instance—can have beneficial effects (Figure 1). Commensal microorganisms exhibit mechanisms that appear to decrease inflammation. They combat pathogenic bacteria by producing bactericidal substances and competing with pathogens and toxins for adherence to the intestinal epithelium. Many regulate immune responses by enhancing innate immunity and modulating signaling pathways and others regulate intestinal epithelial homeostasis by promoting intestinal epithelial cell survival, enhancing barrier function, and stimulating cell protective responses [26]. Clinically, there have been a number of studies and meta-analyses showing a benefit of probiotics in a range of gastrointestinal diseases including pouchitis [27], infectious diarrhea [28,29], and chronic constipation [30]. Most of these studies have been small with methodological limitations. Due to differences in study composition, probiotic doses, and biological activity between various commercial probiotic preparations, results from any given study cannot be applied universally to all probiotic substances. Currently, no probiotic strategy represents standard of care or primary treatment for these GI issues. Nevertheless, given their potential benefit and anti-inflammatory properties, it stands to reason that probiotics may also have a beneficial effect in the prevention and treatment of cancer particularly with regard to preventing or alleviating the adverse effects of traditional cancer therapies. 

## 3. Correlation between the Microbiome and the Development of Colorectal Cancer

**Figure 2:**
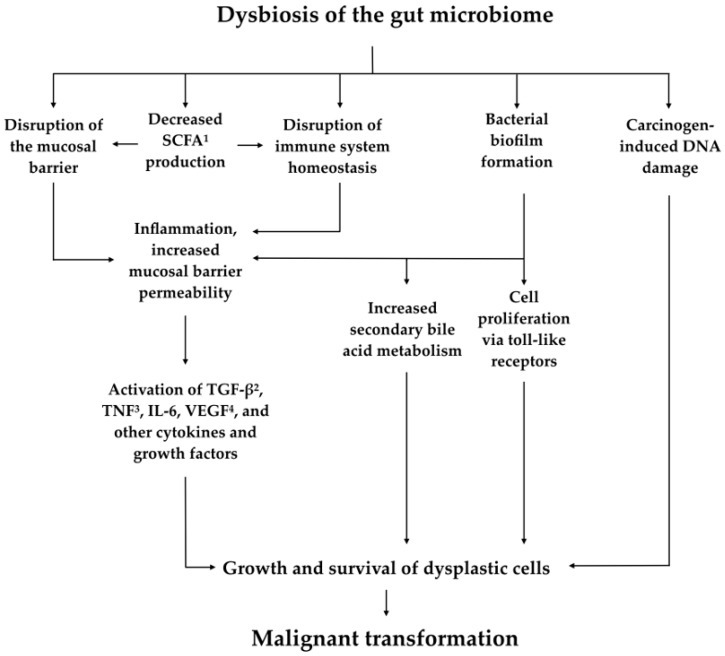
Overview of proposed mechanisms linking dysbiosis of the gut microbiome to tumorigenesis. ^1^ Short chain fatty acids. ^2^ Tumor growth factor beta. ^3^ Tumor necrosis factor. ^4^ Vascular endothelial growth factor.

Recent research has shown that the composition of the microbiome in patients who have developed colorectal cancer is significantly different from the microbiome of patients who have not. A Japanese study that analyzed the micro-genome using terminal restriction fragment length polymorphism and next-generation sequencing analyses noted that several bacterial genera (*Actinomyces*, *Atopobium*, *Fusobacterium*, and *Haemophilus*) as well as several individual species (including *Bacteroides fragilis* and *Streptococcus gordonii*) were significantly associated with patients with known colorectal carcinoma but not in control patients without the disease [31]. Another study noted that microbiota close to the tumor tissue did not differ significantly when compared to the microbiota close to the normal mucosa in the same individual, which suggests that the microbiome does not change secondary to neoplasia but instead that colorectal cancer-distinctive microbiota may be present in the early stages of carcinogenesis [32]. We also know that dysbiosis—an imbalance or maladaptation of the microbiota—affects certain pathways that theoretically can lead to tumorigenesis [12] (Figure 2). Dysbiosis in the GI tract can lead to disruption of homeostasis of the immune system and mucosal barrier with subsequent inflammation resulting in increased mucosal barrier permeability and a continuous state of inflammation. This, in turn, can activate cytokines and growth factors such as tumor growth factor beta (TNF-β), tumor necrosis factor (TNF), IL-6, and vascular endothelial growth factor (VEGF), which can potentially lead to the growth and survival of dysplastic cells [33]. Dysbiosis with bacterial biofilm formation can also lead to increased bile acid metabolism, cell proliferation via Toll-like receptors, and other mechanisms that may, in turn, contribute to malignant transformation [34].

Research into two types of bacteria called *Bacteroides fragilis* and *Fusobacterium nucleatum* provides models for how specific changes in the microbiome can contribute to tumorigenesis. A 2009 study examined enterotoxigenic *B. fragilis* (ETBF) and a cause of acute inflammatory diarrheal disease that also asymptomatically colonizes 20% to 35% of adults [35]. Both EBTF and nontoxigenic *B. fragilis* chronically colonized multiple intestinal neoplasia (Min) mice in the study, but only EBTF was shown to cause colitis and a strong induction of colonic tumors. It was found that ETBF activates the signal transducer and activates transcription-3 (Stat3) in the colon, which leads to colitis characterized by a T helper type 17 (T_H_17) immune response. Antibody blockade of IL-17 and receptor of IL-23—key cytokines in the T_H_17 response—was shown to inhibit the ETBF-induced colitis, subsequent colonic hyperplasia, and tumor formation. 

*Fusobacterium*, particularly *F. nucleatum*, was shown to be over-represented in the genetic analysis of colorectal cancer tissue in two separate 2012 studies [36,37]. Prior to that, the species was linked to periodontitis and appendicitis but not to cancer. It was known that *F. nucleatum* could elicit a pro-inflammatory response, but it was initially unclear whether the bacteria contributed to tumorigenesis or if it was simply an opportunistic infection at the immunocompromised tumor site. Later on, mouse studies confirmed that *F. nucleatum* did, in fact, contribute to colorectal tumorigenesis and contribute to the stimulation of tumor cell growth by activating β-catenin signaling and inducing oncogenic gene expression by the FadA adhesion virulence factor [38]. 

Further studies on mouse models give us the strongest evidence that previously explored connections between dysbiosis and the development of malignancy are causative and not merely correlative. One study published in 2013 noted that tumor-bearing mice showed a different microbiome profile compared to non-tumor-bearing mice. When gut microbiota from tumor-bearing mice were introduced to and allowed to colonize germfree mice, the previously germfree mice were noted to have significantly increased tumorigenesis compared to mice that were colonized with microbiota from tumor-free mice. Furthermore, manipulation of the gut microbiome with antibiotics in these higher-risk mice resulted in a dramatic decrease in the number and size of tumors [11]. Another study in 2015 came to a similar conclusion by showing that isogenetic mice in a controlled environment exhibited different susceptibility to colorectal cancer depending on their distinct gut microflora profile [39].

## 4. Correlation between the Microbiome and Cancer Treatment Effectiveness

A major challenge in cancer therapy research is determining why certain patients will respond to a particular treatment while other patients with similar epidemiologic and clinical characteristics will not. A recent study has suggested that a patient’s microbiome may play a much bigger role in response to systemic cancer therapy than previously realized. One recent *in vitro* study showed that exposing 5-FU-resistant colorectal cancer cells to *Lactobacillus plantarum* selectively inhibited characteristics of the cells including the expression of certain cancer-specific markers (CD44, 133, 166, and ALDH1). Treating the cells with combined *Lactobacillus plantarum* and 5-FU led to caspase-induced apoptosis and demonstrated further potential anticancer effects by inactivating the cells’ Wnt/β-catenin signaling pathway [40]. In live subjects, mouse models have shown that disrupting the microbiota may decrease the effectiveness of systemic cancer therapies. One study evaluated mice that previously had received chronic treatment with antibiotics or were raised in a sterile environment. They were inoculated with subcutaneous tumors and given treatment with either an experimental immunotherapy (CpG-oligonucleotide) or platinum chemotherapy. Antibiotic-treated and germ-free mice had less cytokine production and very little tumor cell death after treatment with immunotherapy when compared to controls. When treated with platinum chemotherapy, the immune cells of antibiotic-treated and germ-free mice produced fewer of the enzymes necessary for generating reactive oxygen species [14]. A separate mouse study evaluated the relation between cyclophosphamide and microbiota. Cyclophosphamide was shown to induce certain Gram-positive bacteria to translocate from the small intestine to secondary lymphoid organs, which, in turn, stimulated a subset of helper and memory T-cells. Tumor-bearing mice that were antibiotic-treated or germ-free that did not have these specific bacteria showed reduced T-cell stimulation and a reduced tumor response after treatment with cyclophosphamide [41]. 

Other studies in mice have looked more specifically into how immunotherapy is affected by microbiota. The CTLA-4 blockade, for instance, appears to be dependent on T-cell responses specific for *Bacteroides thetaiotaomicron* or *Bacteroides fragilis*. In a 2015 study, it was found that tumors in antibiotic-treated or germ-free mice did not respond to treatment with a CTLA-4 blockade but that this lack of response could be overcome by gavage with *B. fragilis*. Interestingly, the CTLA-4 blockade was also found to be effective if *B. fragilis* polysaccharides were administered or if *B. fragilis*-specific T cells were transferred to the subject, which implies that the treatment’s effectiveness was tied to the bacteria’s stimulatory effect on the immune system and not to the continued presence of the bacteria itself. Furthermore, fecal microbial transplant from humans to mice showed that the treatment of melanoma patients with anti-CTLA-4 antibodies favored the outgrowth of *B. fragilis* with anticancer properties by affecting IL-12-dependent T-helper 1 (TH_1_) immune responses to cancer [16]. A similar study done the same year compared melanoma growth in two groups of mice raised in two different facilities and known to harbor different populations of commensal bacteria. The groups showed differences in spontaneous antitumor immunity and tumor growth that disappeared after the mice from the two groups were housed together. Fecal transfer from mice in the population with less tumor growth to mice in the other population resulted in slower tumor growth as well as enhanced tumor infiltration with CD8 positive immune cells. Furthermore, anti-PD-L1 therapy was shown to be more effective in the lower-risk population of mice. Ribosomal RNA sequencing identified *Bifidobacterium* as being associated with antitumor effects. The researchers noted that oral administration of *Bifidobacterium* alone improved tumor control by the same amount as anti-PD-L1 therapy and that combining the two treatments “nearly abolished tumor outgrowth” in the mice [17]. 

While the results of these studies certainly cannot be directly applied to humans, they suggest that the effectiveness of many cancer therapies likely rely, at least in part, on the function of the microbiome. It is further speculated that this may mean the use of certain antibiotics may dampen the effect of certain anti-cancer therapies [15]. Research in human models including a prospective cohort study evaluating whether microbiota composition is correlated with the effectiveness of chemotherapy in colorectal cancer is ongoing [13]. 

## 5. Manipulation of the Microbiome as a Component of Cancer Treatment 

If the microbiome plays such an important role in the development and treatment of colorectal cancer, does that mean we can alter the microbiome to benefit patients? Current research has approached this question in several different ways (Table 1). Specifically—can manipulating the microbiome be used as a means to help prevent cancer from developing? Can it help prevent or alleviate the adverse effects of therapy for patients who have already been diagnosed? In addition, can it help improve the oncological outcomes such as overall survival?

### 5.1. Cancer Prevention

There have been a number of studies in both mouse and human models exploring whether manipulation of the microbiome can prevent cancer development. In colorectal cancer specifically, probiotics are reported to have several cancer-preventative mechanisms that have been shown to operate via intraluminal and systemic effects as well as directly on the intestinal mucosa [49]. In these studies, probiotics have been shown to competitively exclude pathogenic intestinal flora [50,51], alter intestinal microflora enzyme activity [52], reduce carcinogenic secondary bile acids [53], bind carcinogens and mutagens, and increase short chain fatty acid production. Probiotics have also been shown to decrease DNA damage [54] at the level of the intestinal mucosa and help maintain an intestinal barrier function [55]. 

Several studies have looked into the role of probiotics in the prevention of colon cancer and have shown that they appear to have an inhibitory effect on the development of tumors and precancerous lesions even though the effect is not entirely consistent across studies [56]. In one randomized, double-blind, placebo-controlled trial, patients with colon cancer and polypectomized patients were administered a synbiotic consisting of oligofructose-enriched inulin, *Lactobacillus rhamnosus GG*, and *Bifidobacterium lactis Bb12* [57]. Biomarker testing, toxicity assays, and colorectal tissue biopsy samples were checked over the 12-week course of the study with favorable results. Synbiotic administration was associated with reduced colorectal proliferation, reduced capacity of fecal water to induce necrosis in colonic cells, and an improved epithelial barrier function. Colon cancer patients were also noted to have increased the production of interferon gamma and assays performed on colon biopsy samples in polypectomized patients showed decreased exposure to genotoxins. Currently, there is very limited human data directly evaluating colorectal cancer risk in relation to specific characteristics or manipulation of the microbiome. A 2005 trial evaluated colorectal tumor recurrence in 398 men and women with a history of removing at least two prior colorectal tumors who were divided into groups receiving a dietary fiber supplement, *Lactobacillus casei*, both, or neither [43]. While there was no significant difference in the development of new colorectal tumors between the groups, the occurrence rate of tumors with moderate or higher-grade atypia was significantly lower in the group receiving *L. casei*. In addition, a large Italian prospective cohort trial showed that self-reported yogurt intake had an inverse association with colorectal cancer risk (HR 0.65 [95% CI, 0.48–0.89] between the highest and lowest tertile) after accounting for potential confounding factors such as BMI, smoking, and physical activity [44]. While these studies are a long way from showing any clinical role for probiotics in colorectal cancer prevention, they suggest that further study may hold promise.

### 5.2. Alleviating Adverse Effects

Given that previous studies have shown a symptomatic benefit in benign gastrointestinal conditions such as pouchitis and infectious diarrhea, it stands to reason that probiotics may be beneficial in mitigating the adverse gastrointestinal effects of cancer treatment. Mouse studies have supported this hypothesis. For instance, one 2015 study showed that mice treated with intraperitoneally-injected 5-FU developed diarrhea, but their symptoms were improved when given a probiotic suspension. These mice demonstrated the repairing of damage in the jejunal villi as well as reduced mRNA expression of TNF-alpha, IL-1 beta, and IL-6 in intestinal tissue [57]. In another study, mice given with the probiotic mixture VSL#3 had reduced the severity of weight loss and diarrhea after administration of irinotecan as well as reduced apoptosis in the small and large intestine on histological examination [58]. 

Probiotics also seem to help alleviate non-GI adverse effects as well. A separate study looked at the effect of administering *L. casei* and *L. rhamnosus* strains to mice receiving cyclophosphamide. Mice receiving probiotics demonstrated increased immature myeloid progenitors in bone marrow with early recovery of neutrophils in the peripheral blood, improved phagocytic cell recruitment to infectious sites, and increased resistance to opportunistic infection with *Candida albicans* [59]. 

There is limited human data evaluating whether probiotics can help improve tolerance to chemotherapy. In one randomized control trial, colorectal cancer patients starting treatment with irinotecan were concurrently given either placebo or a probiotic containing 10 different strains of bacteria. Unfortunately, the trial was discontinued prematurely due to slow accrual with only 46 out of 220 planned patients enrolled. While the study did show a trend toward reduced frequency of severe diarrhea and use of antidiarrheal drugs in the group receiving probiotics, the study was very limited due to its size [20]. A 2007 clinical trial evaluated 150 patients randomly assigned to receive one of two 5-FU-based chemotherapy regimens (Mayo regimen versus simplified de Gramont regimen) and further randomized to receive or not receive *Lactobacillus rhamnosus GG* with fiber as a supplement during chemotherapy [45]. In total, 26% of the patients received pelvic radiation therapy. Patients receiving *Lactobacillus* had a lower incidence of grade 3 or grade 4 diarrhea (22% vs. 37%, *p* = 0.027), less abdominal discomfort, and had fewer hospitalizations and chemotherapy dose reductions due to bowel toxicity. However, the study was limited due to lack of blinding and placebo control. 

Probiotics have also been studied in the setting of radiation therapy and surgery for the treatment of cancer. In a 2009 meta-analysis of four studies evaluating probiotics in the treatment of radiation enteritis, some of the individual studies showed a significant effect, but the meta-analysis did not demonstrate an overall benefit when all trials were taken into account [46]. Nevertheless, research has shown that intestinal bacteria contribute to radiation-induced injury and repair [60]. Given the variation of probiotic strains and doses between studies, it would be premature to rule out probiotics as a possible treatment in the current state of research. 

Results have been mixed in trials evaluating probiotics as an adjunctive treatment to surgery as well. In one study of 168 patients evaluated after colorectal surgery for cancer, those receiving a four-probiotic regimen had a significantly decreased rate of all postoperative major complications when compared to the placebo arm (28.6% vs. 48.8%, *p* = 0.010) [21]. However, a separate randomized trial evaluating 73 patients who received a prebiotic, a synbiotic, or a standard bowel prep prior to resection for colorectal cancer found no difference in the systemic inflammatory marker response and no effect on post-operative course or complication rates [18]. 

### 5.3. Cancer Outcomes

Data evaluating probiotics as a potential means to prevent cancer or to alleviate the adverse effects of cancer treatment is limited, but data evaluating whether probiotics affect cancer treatment outcomes is even scarcer. Currently, there does not appear to be any published randomized control trials in the literature evaluating whether manipulation of microbiota in patients receiving treatment for colorectal cancer can affect outcomes such as the objective response rate or progression-free survival. Two randomized control trials, however, did evaluate the effect of probiotics on cancer outcomes in bladder cancer. The first, which is a 1995 trial, evaluated 138 patients with primary bladder tumors treated with transurethral resection of the bladder tumor (TURBT). Afterwards, the patients were treated with either an oral preparation of *Lactobacillus casei* or placebo. Patients receiving probiotics had a significant decrease in cancer recurrence with a corrected cumulative recurrence-free rate at one year of 79.2% versus 54.9% with placebo (*p* = 0.01) [59]. A later trial in 2008 evaluated 207 patients with superficial bladder cancer. Patients were treated with TURBT followed by transurethral epirubicin with one group of 100 patients receiving an oral *Lactobacillus casei* preparation once daily for one year. Three-year recurrence free survival was significantly higher in the group receiving probiotics (74.6% vs. 59.9%, *p* = 0.02). However, overall survival did not differ between the two groups [48]. While it is difficult to draw generalized conclusions from these trials, they do suggest that further clinical study would be reasonable especially given the relative safety [61] of administering probiotics. 

## 6. Discussion

We currently have only a very primitive understanding of the microbiome and how it functions in the human body. There is an ongoing debate over even the fundamentals of the microbiome. We know that it is vital to human health, but we are far from being able to define what a “healthy” human microbiome looks like. We know that certain functions such as regulation of the immune system and gut mucosal barrier are beneficial, but it stands to reason that there are a vast number of critical microbiome functions that have not yet been discovered. 

Luckily, we do not need a perfect understanding before translating knowledge of the microbiome into clinical interventions. Studies so far have suggested that restoring function to the microbiome can have beneficial effects in the prevention of cancer and in improving the effectiveness and safety of cancer treatment. As we have seen, administration of certain *Lactobacillus* and *Bifidobacterium* strains is associated with biochemical and histologic changes that may decrease the risk of developing malignancy [50,51]. Other *Lactobacillus* [40], *Bifidobacterium* [17], and *Bacteroides* [16] strains seem to play critical roles in the effectiveness of certain cytotoxic and immune therapies. Nonetheless, the bulk of research in these topics has been in animal models. Data from human research, particularly in clinical trials evaluating whether probiotics or synbiotics can alleviate the adverse effects of various cancer therapies, has so far shown mixed results.

These mixed results are not necessarily unexpected given the early state of clinical research into probiotics, colorectal cancer, and the microbiome. Colorectal cancer provides many different populations, cancer treatment modalities, and patient outcomes to study, which means a probiotic regimen showing a benefit in one cohort will not necessarily show a benefit in all colorectal cancer patients. Several of the clinical trials evaluating probiotics in cancer patients suffer from major limitations ranging from lack of blinding and a placebo control group [56] to inadequate patient accrual [20]. While some of these studies suggest that probiotic therapy is helpful, it will take further clinical research with larger, more diverse patient populations to determine if there is true benefit.

In addition, while some individual probiotic formulations have been studied across multiple clinical trials, there is no strong evidence favoring one formulation over another. Certain bacterial species seem to provide specific helpful functions, but translating this knowledge into clinically beneficial therapies will likely prove difficult. For instance, as previously noted in mouse studies, commensal *Bifidobacterium* seems to be important in the effectiveness of anti-PD-L1 therapy [17]. If this were experimentally proven in humans as well, many questions would remain. Would we be able to overcome a *Bifidobacterium* “deficiency” through the administration of oral probiotics or synbiotics? If so, is there a minimum dosage in colony forming units (CFUs) that would need to be administered for the treatment to be effective? Could other, non-*Bifidobacterium* strains of commensal bacteria perform equivalent functions that amplify the effectiveness of anti-PD-L1 treatments? 

Additionally, what is the benefit, if any, of administering multiple strains of probiotics in a single formulation? There are innumerable interactions between individual bacterial strains in the gut microbiota and certain microbiome compositions appear to make individuals more or less susceptible to colonization with certain pathogenic bacteria [62]. It stands to reason that multiple types of bacteria with anti-tumor effects may synergize when administered together. In fact, many of the clinical trials reviewed in this article examined probiotic formulations instead of probiotics consisting of a single strain. Presumably using probiotic strains with different beneficial mechanisms of action may elicit a better outcome than any one alone and there may be other mechanisms by which commensal bacteria cooperate with each other. However, it is also entirely possible that the opposite is true and giving certain probiotics in combination will cause them to limit each other’s effectiveness.

Ultimately, in order to realize the promise of microbiome-related therapies, research will need to focus on developing defined probiotic therapy regimens with specific strains and doses that may result in a reproducible clinical benefit. Even then, we will need additional research to determine whether a synergistic effect between probiotics and anti-cancer drugs will translate into improved oncologic outcomes such as progression-free or overall survival. 

## 7. Conclusions

Studies evaluating the gut microbiome have shown promising connections between it and the pathogenesis and treatment of colorectal cancer. Further translational research and clinical trials in human subjects are warranted to investigate the possibility of manipulating the microbiome to improve outcomes in colorectal cancer.

## Figures and Tables

**Table 1:** Human studies evaluating probiotics in cancer prevention and treatment.

Study Name	Study Type	Population	Intervention/Cohort Arms	Summary of Key Results
Studies evaluating probiotics and cancer prevention:				
**Rafter 2007** [42]	RCT ^1^	Colon cancer (*n* = 37) & polypectomized (*n* = 43) patients	SYN1 ^2^ + LGG ^3^ + BB12 ^4^ vs. placebo	Several CRC ^5^ biomarkers altered favorably (e.g., decreased genotoxin exposure, IL-2 ^6^, and IFNγ ^7^)
**Ishikawa 2005** [43]	RCT	Tumor-free patients with history of ≥2 colorectal tumors removed (*n* = 398)	Wheat bran vs. *Lactobacillus casei* vs. both vs. neither	No significant difference in colorectal tumor occurrence rate with wheat bran or *L. casei*. However, atypia of tumors was lower in the *L. casei* group.
**Pala 2011** [44]	Prospective cohort study	EPIC-Italy cohort (*n* = 45,241)	Yogurt intake by tertile ^8^	CRC occurrence was significantly lower in highest vs. lowest tertile of yogurt intake. HR ^9^ = 0.62 (95% CI ^10^, 0.46–0.83).
Studies evaluating probiotics and alleviating adverse effects of cancer therapy:				
**Mego 2015** [20]	RCT	CRC patients starting treatment with irinotecan-based therapy (*n* = 46) ^11^	Colon Dophilus ^TM^ probiotic formula vs. placebo	Reduced incidence in probiotic group of severe diarrhea (0% vs. 17.4%, *p* = 0.11) and diarrhea overall (39.1% vs. 60.9%, *p* = 0.11), but not statistically significant.
**Osterlund 2007** [45]	RCT	Post-resection CRC patients requiring adjuvant chemotherapy (*n* = 150)	Randomized to 5-FU via Mayo regimen vs. de Gramont regimen, then randomized to LGG vs. no probiotic	Less grade 3–4 diarrhea in patients receiving LGG (22% vs. 37%, *p* = 0.027)
**Fuccio 2009** [46]	Meta-analysis	Three RCTs evaluating probiotic supplementation to prevent radiation induced diarrhea (*n* = 632). One RCT evaluating therapeutic role.	Probiotic supplementation vs. placebo/control	No significant difference in rates of radiation-induced diarrhea between probiotic and control arms in preventative trials (OR ^12^ 0.47, 95% CI 0.13–1.67) or in the single therapeutic trial
**Kotzampassi 2015** [21]	RCT	Patients undergoing surgery for CRC (*n* = 168) ^13^	Probiotic formulation ^14^ vs. placebo	Significant decrease in all major post-operative complications in probiotics arm (28.6% vs. 48.8%, *p* = 0.010, OR 0.42)
**Krebs 2016** [18]	RCT	Patients undergoing surgery for CRC (*n* = 73)	Preoperative prebiotics ^15^ vs. preoperative synbiotics ^16^ vs. mechanical bowel cleansing	No statistical difference in systemic inflammatory response, postoperative course, or complication rate
Studies evaluating probiotics and cancer outcomes:				
**Aso 1995** [47]	RCT	Patients with superficial transitional cell carcinoma of the bladder after transurethral resection (*n* = 138)	Oral *L. casei* preparation vs. placebo	Reduced recurrence rate in patients with primary multiple tumors or recurrent single tumors (*p* = 0.01), but not with recurrent multiple tumors
**Naito 2008** [48]	RCT	Patients with superficial bladder cancer after transurethral resection and intravesicular epirubicin (*n* = 202)	Intravesicular epirubicin plus *Lactobacillus casei* vs. epirubicin alone	Three-year recurrence-free survival rate was significantly higher in the group receiving *L. casei* (74.6% vs. 59.9%, *p* = 0.0234), no difference in PFS ^17^ or OS ^18^

^1^ Randomized control trial. ^2^ Synbiotic preparation-oligofructose-enriched inulin. ^3^ *Lactobacillus rhamnosus GG*. ^4^ *Bifidobacterium lactis Bb12*. ^5^ Colorectal cancer. ^6^ Interleukin-2. ^7^ Interferon gamma. ^8^ As assessed by a dietary questionnaire. ^9^ Hazard ratio. ^10^ Confidence interval. ^11^ Study prematurely terminated due to slow accrual. ^12^ Odds ratio. ^13^ Study prematurely stopped due to efficacy in the primary outcome. ^14^ Consisting of *Lactobacillus acidophilus*, *L. plantarum*, *Bifidobacterium lactis*, and *Saccharomyces boulardii*. ^15^ Consisting of betaglucan, inulin, pectin, and resistant starch. ^16^ Consisting of the prebiotic formulation plus *Pediacoccus pentosaceus* 5–33:3, *Leuconostoc mesenteroides* 32–77:1, *Lactobacillus casei* subspecies *paracasei* 19, and *Lactobacillus plantarum* 2362. ^17^ Progression free survival. ^18^ Overall survival.
